# Evaluation of the effects of β1-selective beta-blockers on bone mineral density and fracture risk in postmenopausal women

**DOI:** 10.3906/sag-1909-187

**Published:** 2020-06-23

**Authors:** Betül YAVUZ KELEŞ, Meltem VURAL, Burcu ÖNDER, Kadriye ÖNEŞ

**Affiliations:** 1 İstanbul Physical Medicine and Rehabilitation Training and Research Hospital, İstanbul Turkey; 2 Bakırköy Dr. Sadi Konuk Training Hospital, İstanbul Turkey

**Keywords:** Adrenergic beta-antagonists, postmenopause, bone mineral density

## Abstract

**Background/aim:**

β1-selective beta-blockers (BBs) are sympatholytic agents, and discerning their effects on bone health would be of great importance. This study aimed to investigate the influence of β1-selective BBs on bone mineral density (BMD) and fracture risk.

**Materials and methods:**

This study included postmenopausal women who used β1-selective BBs (BB group) and control group. Sociodemographic characteristics, BMD and previous fragility fractures were recorded. Additionally, the 10-year probability of a major osteoporotic and hip fracture was calculated using the fracture risk assessment tool (FRAX).

**Results:**

A total of 60 participants were included in the study. L1-4 and L2-4 BMD values were significantly higher in BB group than control group (P = 0.015 and P = 0.025, respectively). Moreover, T-scores of lumbar and femur total were significantly higher in the BB group. Two patients in BB and 6 patients in control group had previous fragility fracture. No statistically significant intergroup difference was noted regarding FRAX.

**Conclusion:**

Based on our results, β1-selective BB usage was associated with higher BMD at the lumbar region in postmenopausal women.

##  1. Introduction

Osteoporosis is a disease with worldwide prevalence. It decreases bone mass and strength, thereby resulting in fractures and primarily affects postmenopausal women [1–3]. Almost, 40% postmenopausal women have osteoporosis [4]. A deterioration in bone mass and strength with aging is an expected situation besides the several factors that may affect bone mass and strength, such as inflammation, endocrinological and metabolic disorders and some drugs [5–7].

Recent studies have shown that the sympathetic nervous system significantly affects bone metabolism [8–12]. Typically, sympathetic activation is considered to contribute to bone loss [13]. This effect was believed to be mainly mediated by β2 adrenergic receptors [9–11]. However, some researchers have shown that β1 and β3 adrenergic receptors are also present in the human periosteal osteoblasts and human osteosarcoma-derived cells [14].

Beta-blockers (BBs), especially β1-selective BBs, are the commonly used sympatholytic agents and find use in several cardiovascular diseases such as hypertension, arrhythmias, myocardial infarction, congestive heart failure and angina pectoris [15]. Osteoporosis and cardiovascular diseases often occur together during the postmenopausal period [16]. Several studies have investigated the effects of nonselective BBs on fracture risk and bone mineral density (BMD), albeit with conflicting results [16–20]. In contrast, some researchers have found β1 receptors in the human periosteal osteoblasts, which makes it imperative to determine the effects of β1-selective BBs on bone health. In a previous study, it was concluded that metoprolol prevented the bone loss in ovariectomized rats [21]. 

This study aimed to investigate the effects of β1-selective BBs on BMD, fragility fracture and the 10-year predicted probability of major osteoporotic and hip fractures. To our knowledge, no investigation has yet been done regarding the 10-year predicted probability of major osteoporotic and hip fractures in postmenopausal women using β1-selective BBs.

## 2. Materials and methods 

### 2.1. Study design and subjects

This cross-sectional study has been approved by the local ethics committee of Bakırköy Dr. Sadi Konuk Training Hospital (2018/57) and has been performed per the ethical standards laid down in the 1964 Declaration of Helsinki and its later amendments. Informed consent was obtained from the subjects.

Postmenopausal women who were admitted to the outpatient clinic İstanbul Physical Medicine and Rehabilitation Training and Research Hospital between January 2018 and September 2018 were evaluated for this study. Women who had not menstruated for at least 1year were considered menopausal as per the definition of the World Health Organization [22].

Criteria for exclusion were as follows: Older than 65 years, abnormality in laboratory tests (complete blood count, serum creatinine, calcium, phosphorus), endocrine disorders, chronic liver disease, history of steroid use (at any time more than 3 months), anticonvulsant and anticoagulant drug use, acute and chronic infectious disease, inflammatory disease, history of malignancy, secondary osteoporosis and undergoing treatment for osteoporosis. Because of the potential impact on bone turnover, patients who used a drug for more than 3 months besides β1-selective BBs were excluded from the study. The study comprised 2 groups: BB and control groups. The control group included postmenopausal women who were age- and menopause duration-matched with those of the BB group and had no history of BBs usage. The minimum BB usage period was determined as 6 months in the BB group and all women in BB were using β1-selective BBs when included in the study.

### 2.2. Methods

#### 2.2.1.Demographic characteristics

Age (year), height (cm), weight (kg), body mass index (BMI, kg/m2) and time after the last menstruation cycle (year) of the participants were recorded. Additionally, the participants were also asked about their smoking status, alcohol intake, regular exercise, previous fragility fracture with the timing of the fracture (premenopausal or postmenopausal) and history of hip fracture in the family. Fractures that occurred during childhood were not recorded. An exercise regime of more than 30 min at least thrice a week was considered as regular exercise. The period of β1-selective BBs use was recorded for patients of BBs group per the information received from them.

#### 2.2.2. Bone mineral density

The BMD was measured at the lumbar spine and femur by using dual-energy X-ray absorptiometry (DPX-LUNAR 74400). Lumbar spine (L1-4;L2-4), femur neck and femur total T-scores and BMD (g/cm2) were recorded.

#### 2.2.3. Fracture risk assessment tool (FRAX)

FRAX calculates the 10-year fracture probability of a major osteoporotic (FRAX-major) and hip fracture (FRAX-hip) by using some variables (age, BMI, previous fracture, parent hip fracture, current smoking, glucocorticoid use, alcohol consumption, rheumatoid arthritis and secondary osteoporosis). FRAX-hip and FRAX-major of subjects were calculated using the Turkish version of the FRAX assessment tool [23,24].

### 2.3. Sample size

Power analysis was performed with reference to a previous study to determine the minimum number of participants planned to be included in the study [25]. Minimum 26 participants for each group were needed to achieve the effect size (d = 0.84) of the previous study with alfa error probability of 0.05 and 80% power.

### 2.4. Statistical analysis

Statistical studies were performed using the MedCalc software, version 18.11.3. Descriptive statistics were shown as the mean ± standard deviation for continuous variables, and nominal variables were presented as the number of cases and percentages (%). Normality was tested by Kolmogorow-Smirnov test for continuous variables. Intergroup comparisons of the various parameters were performed using the independent-samples t-test and the chi-square test wherever applicable. Pearson method was used to calculate the correlation rho. A value of P < 0.05 was considered statistically significant.

## 3. Results

This study evaluated 215 postmenopausal women, of which 60 women who met the study criteria were included. The BB and control group each comprised 30 subjects with a mean age of 57.86 ± 5.66 in the BB group and 55.41 ± 5.9 in the control group. Mean cessation of menstruation was 9.14 ± 5.73 years in BB group and 9.45 ± 6.96 years in the control group. The average period of β1-selective BBs use was 42.6 ± 36.7 months. No statistically significant difference was found in the mean age, cessation of menstruation, weight, height, BMI, smoking and regular exercise. Alcohol consumption was absent in both groups. There were 2 subjects in the BB group and 6 in the control group who had a previous fracture (P = 0.132). The previous fractures noted in the BB group were that of the foot and costa. The control group had prior fractures in the hip, proximal tibia, proximal humerus, foot and two in the forearm. All these fractures occurred during the postmenopausal period. The fractures in the BB group occurred during BB usage. The demographic and general features of each group are shown in Table 1.

**Table 1:** The demographic features of the groups.

	BBs group(N = 30)	Control group(N = 30)	P
L1-4 BMD (g/cm2)	0.9±0.2	0.8 ± 0.1	0.015*
L2-4 BMD (g/cm2)	0.9 ± 0.2	0.8 ± 0.1	0.025*
Femur neck BMD (g/cm2)	0.8 ± 0.0	0.8 ± 0.1	0.296
Femur total BMD (g/cm2)	0.9 ± 0.0	0.9 ± 0.1	0.065
L1-4 T-score	-1.4 ± 1.3	-2.1 ± 0.7	0.020*
L2-4 T-score	-1.3 ± 1.4	-2.1 ± 0.7	0.016*
Femur neck T-score	-0.5 ± 0.9	-0.9 ± 0.7	0.141
Femur total T-Score	-0.4 ± 0.6	-1 ± 0.8	0.006*
Frax-major fracture (%)	4.7 ± 2.0	5.3 ± 2.7	0.412
Frax-hip fracture (%)	0.5 ± 0.4	0.9 ± 0.8	0.078

*P < 0.05. Values are expressed as mean ± standard deviation,

Lumbar spine (L1-4 and L2-4) BMD values were significantly higher in BB group compared with the control group (P = 0.015 and P = 0.025, respectively). Additionally, T-scores of lumbar spine (L1-4 and L2-4) and femur total were significantly higher in BB group (P = 0.02, P = 0.016 and P = 0.006, respectively) compared with the control group. No statistically significant intergroup difference was found regarding the 10-year fracture probability of a major osteoporotic and hip fracture. Table 2 shows the comparison of BMDs, T-scores, FRAX-major and FRAX-hip of the 2 groups.

**Table 2:** The demographic features of the groups.

	BBs group(N = 30)	Control group(N = 30)	p
Age (years)	57.9 ± 5.7	55.4 ± 5.9	0.113a
Weight (kg)	75.6 ± 9.9	69.9 ± 2.2	0.097 a
Height (cm)	154.8 ± 5.5	156.2 ± 4.5	0.366 a
BMI (kg/m2)	31.4 ± 4.3	28.7 ± 4.4	0.056 a
Cessation of menstruation (years)	9.1 ± 5.7	9.4 ± 6.9	0.857 a
Smoking (%)	10	20	0.138 b
Alcohol (%)	0	0	
Previous fracture (n)	2	6	0.132 b
Regular exercise (%)	16.6	3.3	0.087 b

BMI: Body mass index, (mean ± SD), a Independent-samples t-test, b Chi-square test. *P < 0.05.

## 4. Discussion 

In this study, we investigated the effects of β1-selective BB use on bone health in postmenopausal women. Based on the study results, β1-selective BB use was associated with higher BMD in the lumbar region**. **Additionally, the lumbar and femoral total T-scores were better in the BB group compared with the control group.

Farr et al. showed that sympathetic activity was 2–4 -fold higher in postmenopausal women than in premenopausal women [26]. Furthermore, evidence suggests that sympathetic activity has a deleterious effect on bones [27]. Takeda et al. showed that leptin regulates bone formation through the sympathetic nervous system and the systemic administration of nonselective BBs increases bone mass [8]. Furthermore, they reported that leptin affects the osteoblasts through β2 adrenergic receptors. Subsequent studies have shown the presence of β1 and β3 adrenergic receptors in the bone [14].

Zhang et al. investigated the effects of metoprolol (β1-selective BB) on osteoblasts in ovariectomised rats. They concluded that metoprolol prevents oestrogen deficiency-induced bone loss and recommended metoprolol as an alternative treatment for postmenopausal osteoporosis in hypertensive patients [21]. Furthermore, Khosla et al. showed that the trabecular bone volume and trabecular number were significantly better in the patients using β1-selective BB group, but there was no significant difference in cortical bone parameters [28]. Concordant with this result, our BB group showed better BMD values in the lumbar region where the trabecular bone is predominant. In contrast, no significant difference was detected in the BMD and T-score of the femoral neck where the cortical bone is predominant. Another study has also shown a negative correlation between sympathetic activity and trabecular bone [21].

In this study, we also evaluated the 10-year probability of a major osteoporotic and hip fracture in participants by using FRAX. Based on our results, the mean value of the 10-year predicted probability of major fracture was 4.69% in the BB group and 5.35% in the control group. Even though the mean value of major hip fracture risk was 0.48% in the BB group and 0.87% in the control group, the intergroup difference was not statistically significant. Tuzun et al. found the mean 10-year major osteoporotic fracture probability to be 6.0% and that of hip fracture to be 2% in Turkish population aged 50 years or more [29]. The major osteoporotic and hip fracture probabilities in postmenopausal women were determined as 5.5% and 0.9%, respectively, per a previous study result, which is similar to the FRAX results of our control group [30].

Furthermore, there were 2 subjects with a previous fragility fracture in the BB group and 6 subjects in the control group. Considering this result, the fracture rate in the control group seems slightly higher, but the intergroup difference was not statistically significant. The findings of a meta-analysis that investigated the effects of BBs on fracture suggested that the risk of fracture is about 15% lower in patients treated with BB and this effect has been mostly attributed to β1-selective BB by authors [15].

To our knowledge, the 10-year predicted probability of major osteoporotic and hip fracture was not investigated before in postmenopausal women using β1-selective BBs, which is a significant strength of our study. Moreover, we excluded the subjects who took other drugs besides BBs to eliminate their effects on bone, which is another advantage of the present study. Even though β1-selective BBs are commonly used drugs, it was some hard to find the subjects who only used β1-selective BBs without other cardiovascular and other medications because we wanted to evaluate only the effects of β1-selective BB on the bone. Notably, most of the women using β1-selective BBs were also taking other drugs.

Nonetheless, this study has some limitations. First, this was a cross-sectional study. Therefore, further prospective, randomised, controlled studies in the future may provide health professionals with more information regarding fracture risk and BMD in subjects using β1-selective BBs. Second, subjects who regularly exercised were more likely to be in the BB group, although there was no statistically significant difference. However, this may have positively contributed to BMD.

In conclusion, β1-selective BB usage was associated with higher BMD at the lumbar region in postmenopausal women. The dual beneficial effects of β1-selective BBs on both the cardiovascular and skeletal systems in postmenopausal women can be advantageous for patients who are required to use these drugs for a long time or sometimes for a lifetime.

## Acknowledgments/Disclaimers

This study was presented in Turkish Rheumatology Congress with international participation 2018. The authors received no financial support for the research and/or authorship of this article. The authors would like to thank Enago (www.enago.com) for the English language review.

## Conflict of interest

The authors declared no conflicts of interest with respect to the authorship and/or publication of this article.
